# Exploring the association of vitamin B12 deficiency and dental health in older adults

**DOI:** 10.3389/fnut.2025.1589062

**Published:** 2025-06-18

**Authors:** Man Hung, Amir Mohajeri, Jacob Marx, Corban Ward, Martin S. Lipsky

**Affiliations:** ^1^College of Dental Medicine, Roseman University of Health Sciences, South Jordan, UT, United States; ^2^The Wharton School, University of Pennsylvania, Philadelphia, PA, United States; ^3^Department of Orthopaedic Surgery Operations, University of Utah, Salt Lake City, UT, United States; ^4^Division of Public Health, University of Utah, Salt Lake City, UT, United States; ^5^Huntsman Cancer Institute, Salt Lake City, UT, United States; ^6^Institute on Aging, Portland State University, Portland, OR, United States

**Keywords:** vitamin B12, dental caries, older adults, NHANES, oral health, dental health

## Abstract

**Background:**

Vitamin B12 plays a crucial role in overall health, yet its impact on dental health, particularly dental caries, remains underexplored. Older adults are at an increased risk of vitamin B12 deficiency and dental disease, but the relationship between these two factors is not well understood. This study examines the association between serum vitamin B12 levels and dental health in older adults using data from the NHANES 2011–2014 cycles.

**Methods:**

A cross-sectional analysis was conducted using NHANES data from 1,907 participants aged 65 and older. Serum vitamin B12 levels were categorized as normal (>221 pmol/L), marginal (148–221 pmol/L), and deficient (<148 pmol/L). Dental status was assessed using the Decayed, Missing, and Filled Teeth (DMFT) index based on NHANES dental health examinations. Poisson regression models evaluated associations between vitamin B12 levels and DMFT scores, adjusting for age, sex, race/ethnicity, education, and income.

**Results:**

Lower vitamin B12 levels were significantly associated with higher DMFT scores (*p* < 0.05). Participants with marginal and deficient vitamin B12 levels exhibited increased DMFT scores compared to those with normal vitamin B12 levels, and these differences persisted after adjusting for demographic and socioeconomic factors. The findings suggest that inadequate vitamin B12 status may contribute to a greater cumulative burden of dental disease in older adults.

**Conclusion:**

These findings suggest that vitamin B12 deficiency may contribute to a greater lifetime burden of dental disease in older adults. Further research is needed to explore causal mechanisms and assess whether vitamin B12 supplementation could be a preventive measure for maintaining dental health in aging populations.

## 1 Introduction

Oral health is vital for overall well-being (1) and for its impact on nutrition, physical health, and quality of life (2). The prevalence of poor oral health increases with age, and tooth decay occurs nearly twice as often in older adults than in younger ones (3). Untreated dental caries can lead to severe tooth decay, infection, tooth loss, pain, and systemic complications (4).

The intake of micronutrients and macronutrients is necessary for maintaining the health and integrity of the oral mucosa and dental hard tissues. Proper nutrition prevents gum disease and tooth decay (5), and a deficiency in essential nutrients can compromise oral health, resulting in a negative feedback cycle that adversely affects the teeth, oral mucosa, and periodontium (6).

One key micronutrient is Vitamin B12, a water-soluble vitamin primarily obtained from animal-based dietary sources such as meat, eggs, and dairy products (7). Vitamin B12 is essential for DNA synthesis, red blood cell production, and effective nerve function and communication (8). Older adults are at increased risk of vitamin B12 deficiency due to age-related changes in the gastrointestinal tract that impair absorption, insufficient dietary intake, and medications that affect Vitamin B12 metabolism (9). As a result, vitamin B12 levels tend to decrease with age and vitamin B12 deficiencies are more prevalent among older adults (10).

Additionally, vitamin B12 supports immune responses, which are crucial for protecting the oral cavity from bacterial infections that can lead to gingivitis or periodontitis (11). Recent studies demonstrate the significance of nutrition in oral health, revealing important links between nutritional deficiencies and various oral diseases (12). For example, vitamin D and calcium are essential for maintaining tooth integrity (13), while deficiencies in vitamin C can cause gingival and periodontal disease (14). However, the role of vitamin B12 in dental health is less understood. Theoretically, its deficiency could influence oral health by impairing the immune response and changing the oral microbiome. In addition, suboptimal vitamin B12 levels are associated with reduced bone mineral density (BMD) (15, 16), and a lower BMD is linked to increases in the absent, decayed, missing, and filled teeth (DMFT) index (17, 18). Despite these plausible biological mechanisms and a study linking B-12 to an increased risk of caries in children (19), research exploring vitamin B12 deficiency and dental caries remains limited (20). Since B-12 deficiency becomes more common with age, exploring a possible link between caries among older adults warrants further examination.

This study examined the association between serum vitamin B12 levels and the burden of dental disease among older adults using data from the NHANES 2011–2014 cycle. Specifically, the study sought to determine whether lower vitamin B12 levels are linked to DMFT scores. Given the biological role of vitamin B12 in maintaining oral and systemic health, it is hypothesized, as a directional hypothesis, that individuals with lower vitamin B12 levels will exhibit higher DMFT scores than those with normal B12 levels. Identifying an association might help public health advocates and oral health providers develop strategies to incorporate into oral health care for aging populations.

## 2 Materials and methods

### 2.1 Data source

NHANES is a large-scale, stratified, multi-phase probability survey designed to reflect the health status of the United States (U. S.) civilian, non-institutionalized population. The NHANES data collection process includes structured household interviews, followed by comprehensive clinical assessments conducted at mobile examination units. During these evaluations, biological specimens are obtained for laboratory testing. Before participation, individuals or their legally authorized representatives, provided signed consent forms. The Research Ethics Review Board of the National Center for Health Statistics provides ethical oversight for NHANES data collection. Additional details regarding NHANES methodology are available at the Centers for Disease Control and Prevention website (21).

**Figure 1:**
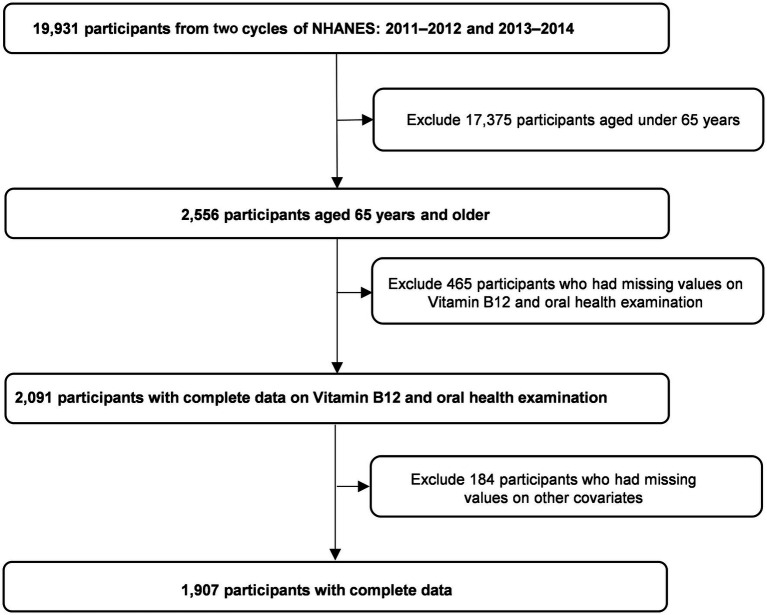
Sample selection criteria flowcharts.

This study utilized data from the 2011–2012 and 2013–2014 NHANES cycles since these included serum vitamin B12 measurements for adults. Participants were eligible for inclusion if they were at least 65 years old (*n* = 2,556) since this age group is a high-risk group for B-12 deficiency and had documented data on dental health status, serum B12 levels, and demographic attributes of interest. Individuals under 65 or with missing information on a key variable were excluded from the analysis (complete-case analysis). An *a priori* sample size calculation was not performed since this was a secondary analysis of existing nationally representative survey data. Figure 1 summarizes participant selection. Ultimately, the study consisted of 1,907 individuals who met the inclusion criteria.

### 2.2 Assessment of variables

Dental health assessments were performed by licensed dental professionals who underwent rigorous NHANES-specific training to ensure standardized examinations. The dental professionals conducted their evaluations in mobile facilities outfitted with a portable examination chair, artificial lighting, compressed air, and digital imaging technology. Dental status was assessed using the DMFT index, which quantifies the total number of decayed, missing, and filled teeth, providing a comprehensive measure of cumulative dental caries experience. According to the ‘Coronal Caries: Tooth Count’ segment of the dental examination, the DMFT index was calculated as the total count of codes E, J, K, M, P, Q, R, T, X, and Z. Code E denotes missing due to dental disease, J indicates the presence of a permanent root tip without restoration or an attached device, K signifies a primary tooth with a carious surface condition, M represents missing due to other causes, P denotes missing due to dental disease but replaced by a removable restoration, Q indicates missing due to other causes but replaced by a removable restoration, R signifies missing due to dental disease but replaced by a fixed restoration, T denotes the presence of a permanent root tip with a restoration or attached device, X indicates missing due to other causes but replaced by a fixed restoration, and Z represents a permanent tooth with a carious surface. Caries scoring criteria used in the dental examination, and quality assurance and training/calibration details, are described in-depth in the NHANES plan and operations manual (22). A quality control protocol involving blinded, repeat evaluations demonstrated strong inter-examiner agreement.

Blood samples were drawn on the same day as the dental examination, and serum B12 levels were determined using an electrochemiluminescence immunoassay. Participants were categorized into three distinct groups based on serum B12 concentration: normal (>221 pmol/L), borderline (148–221 pmol/L), and deficient (<148 pmol/L) (23, 24).

Demographic characteristics such as age, sex, racial/ethnic background, education level, and the ratio of income to poverty (PIR) were included as covariates in the analysis. Sex was classified as either male or female, while racial/ethnic groupings included non-Hispanic White, non-Hispanic Black, non-Hispanic Asian, other Hispanic, Mexican American, and other racial categories. Race/ethnicity was included as a variable because of evidence that ethnicity and race influence homocysteine and vitamin B12 metabolism (25–27). Education level was categorized into five groups: less than 9th grade, 9th–11th grade, high school graduate/GED, some college, and college graduate or above. We categorized PIR into three levels: low income (PIR < 1), middle income (PIR 1–4), and high income (PIR > 4) (28). PIR reflects income relative to household needs and was determined by accounting for annual variations in household size and cost of living while adjusting for the consumer price index using household income and federally established poverty thresholds (29, 30).

### 2.3 Statistical procedures

Mean values and standard deviations (SD) were calculated for continuous variables while categorical variables were expressed as counts and percentages. The analysis employed the Chi-square test to determine statistical differences in categorical data distributions. For continuous variables, we first evaluated the distribution of the DMFT scores using the Shapiro–Wilk test for normality. Since the DMFT scores were not normally distributed (*p* < 0.05), non-parametric tests were used for subsequent analyses. The Mann–Whitney *U* test was used for two-group comparisons, while the Kruskal–Wallis test was applied when more than two groups were analyzed. Correlations between continuous variables were assessed using Spearman’s rank correlation coefficient due to the non-normal distribution of the DMFT data. Dunn-Bonferroni’s multiple comparison post-hoc test was used to identify specific group differences when statistical significance was observed.

To evaluate the association between vitamin B12 status and dental health, Poisson regression models were constructed to examine the relationship between serum vitamin B12 levels and DMFT scores. The results were expressed as rate ratios (RRs) with 95% confidence intervals (CIs). The statistical analysis followed a stepwise modeling approach: Model I provided an unadjusted assessment of the association between B-12 and DMFT scores, while Model II adjusted for age, and racial/ethnic background. Model III further controlled for educational level, and poverty income ratio (PIR). A *p*-value threshold of less than 0.05 was established to determine statistical significance.

## 3 Results

### 3.1 Distribution of DMFT scores

Among the 1,907 older adults included in the analysis, the mean age was 73.33 ± 5.37 years. Mean DMFT scores differed across different demographic and socioeconomic groups. Mexican American and non-Hispanic Asian participants had the lowest DMFT scores (17.89 ± 7.38 and 17.22 ± 8.26, respectively), whereas those categorized as “Other Race” had the highest scores (23.20 ± 6.35, *p* < 0.001) (Table 1).

**Table 1:** Distribution of mean DMFT score by participant characteristics in the NHANES cycles 2011–2014.

Variables	All participants	Participants DMFT score		
		Mean ± SD	*p-*value	*Post hoc* test^*****^
Age (years)	73.33 ± 5.37	73.35 ± 5.37	<0.001^**^ (0.205)	
Sex				
Female	965 (50.6%)	19.83 ± 6.59	0.934^***^	
Male	942 (49.4)	19.84 ± 6.88		
Race and Ethnicity				
Mexican American^a^	137 (7.2%)	17.89 ± 7.38	**<0.001** ^****^	**0.011** ^**a,c**^ **, 0.001** ^**a,f**^ **, 0.042** ^**a,d**^ **, 0.019** ^**b,e**^ **, 0.001** ^**c,e**^ **, 0.004** ^**d,e**^ **, 0.000** ^**e,f**^
Other Hispanic^b^	159 (8.3%)	20.16 ± 6.59		
Non-Hispanic White^c^	1,047 (54.9%)	20.25 ± 6.21		
Non-Hispanic Black^d^	388 (20.3%)	19.98 ± 6.96		
Non-Hispanic Asian^e^	146 (7.7%)	17.22 ± 8.26		
Other Race*^,f^	30 (1.6%)	23.20 ± 6.35		
Education level				
Less than 9th grade^g^	282 (14.8%)	21.25 ± 7.12	**<0.001** ^****^	**0.033** ^**k,j**^ **, 0.000** ^**k, I**^ **, 0.000** ^**k,g**^ **, 0.000** ^**k,h**^ **, 0.003** ^**j, I**^ **, 0.000** ^**j,g**^ **, 0.000** ^**j,h**^
9–11th grade^h^	271 (14.2%)	21.45 ± 6.87		
High school graduate/GED^i^	434 (22.8%)	20.64 ± 6.63		
Some college^j^	505 (26.5%)	19.13 ± 6.47		
College graduate or above^k^	415 (21.8%)	17.84 ± 6.17		
Ratio of Income to Poverty (PIR)				
Low (<1)^l^	337 (17.7%)	21.42 ± 7.13	**<0.001** ^****^	**0.000** ^**n,m**^ **, 0.000** ^**n,l**^ **, 0.001** ^**m,l**^
Middle (1–4)^m^	1,146 (60.1%)	20.10 ± 6.71		
High (>4)^n^	424 (22.2%)	17.87 ± 5.97		
B12 (pmol/L)				
Normal (>221)^o^	1,671 (87.6%)	19.65 ± 6.69	**0.002** ^****^	**0.010** ^**o,p**^
Marginal (148-221)^p^	180 (9.4%)	21.04 ± 6.97		
Deficient (<148)^q^	56 (2.9%)	21.57 ± 6.48		

Bold indicates *p* < 0.05. *Includes multi-racial participants. ^**^Spearman Rank Correlation ^***^Mann–Whitney *U* test. ^****^Kruskal–Wallis test. ^*****^Dunn-Bonferroni’s multiple comparisons test for *post hoc*.

^a–q^ Shows which variables are being compared in the *post hoc* test.

Education level was also associated with DMFT scores. Participants with the lowest education level had the highest DMFT scores (21.25 ± 7.12, *p* < 0.001), while those with a college degree or higher exhibited the lowest scores (17.84 ± 6.17). A similar pattern was observed for income: individuals in the lowest income group had significantly higher DMFT scores (21.42 ± 7.13) compared to those in the highest income group, who had the lowest scores (17.87 ± 5.97) (*p* < 0.001) (Table 1).

*Post hoc* analysis showed significant differences in DMFT scores between several groups. However, no significant differences were found among various race/ethnicity pairs, adjacent education levels, or between B12-deficient individuals and those with marginal or normal B12 levels (all *p* > 0.05) (Table 1).

### 3.2 Serum vitamin B12 levels and participant characteristics

Vitamin B12 deficiency (<148 pmol/L) was identified in 2.9% of the study population, while 9.4% had marginal levels (148–221 pmol/L), and 87.6% had normal levels (>221 pmol/L) (Table 1). Vitamin B12 levels were significantly associated with race/ethnicity, education, and income (*p* < 0.001). Non-Hispanic Asian participants had the lowest prevalence of vitamin B12 deficiency (1.4%), whereas “Other Hispanic” individuals had the highest (5.7%, *p* < 0.001). Education and socioeconomic status were also linked to vitamin B12 deficiency, with lower levels observed in individuals with less than a high school education (3.9%) and those living below the poverty line (3.3%, *p* < 0.001) (Table 2).

**Table 2:** Serum B12 concentrations in participants aged 65 years and older (NHANES 2011–2014) (*N* = 1907).

Characteristics	Normal (>221)	*P*-value/*Post hoc* test^***^	Marginal (148–221)	*P*-value/*Post hoc* test^***^	Deficient (<148)	*P*-value/*Post hoc* test^***^
*N*	1,671	–	180	–	56	–
Sex						
Female	859 (89%)	0.250^**^	80 (8.3%)	0.136^**^	26 (2.7%)	0.593^**^
Male	812 (86.2%)		100 (10.6%)		30 (3.2%)	
Race and Ethnicity						
Mexican American^a^	115 (83.9%)	<0.001^**^/0.003^c,e^	16 (11.7%)	**<0.001** ^**^	6 (4.4%)	**<0.001** ^**^
Other Hispanic^b^	137 (86.2%)		13 (8.2%)		9 (5.7%)	
Non-Hispanic White^c^	914 (87.3%)		104 (9.90%)		29 (2.8%)	
Non-Hispanic Black^d^	345 (88.9%)		33 (8.5%)		10 (2.60%)	
Non-Hispanic Asian^e^	134 (91.8%)		10 (6.8%)		2 (1.4%)	
Other Race*^,f^	26 (86.7%)		4 (13.3%)		-	
Education level						
Less than 9th grade^g^	240 (85.1%)	**<0.001** ^**^	31 (11%)	0.075^**^	11 (3.9%)	0.382^**^
9–11th grade^h^	233 (86%)		30 (11.1%)		8 (3%)	
High school graduate/GED^i^	373 (85.9%)		45 (10.4%)		16 (3.7%)	
Some college^j^	446 (88.3%)		46 (9.1%)		13 (2.6%)	
College graduate or above^k^	379 (91.3%)		28 (6.7%)		8 (1.9%)	
Ratio of Income to Poverty (PIR)						
Low (<1)^l^	286 (84.9%)	<0.001^**^/0.002^l,n^	40 (11.9%)	**<0.001** ^**^	11 (3.3%)	**<0.001** ^**^
Middle (1–4)^m^	990 (86.4%)		119 (10.4%)		37 (3.2%)	
High (>4)^n^	395 (93.2%)		21 (5%)		8 (1.9%)	

Bold indicates *p* < 0.05. *Includes multi-racial participants. ^**^Chi-square test. ^***^Dunn-Bonferroni’s multiple comparisons test for *post hoc*.

^a–n^ Shows which variables are being compared in the *post hoc* test.

*Post hoc* analysis revealed significant differences in serum vitamin B12 levels between Non-Hispanic White and Non-Hispanic Asian participants (*p* < 0.05), as well as between low-income and high-income groups (*p* < 0.05) (Table 2).

### 3.3 Association between vitamin B12 levels and DMFT scores using adjusted models

Table 3 reports the multivariable regression models used to examine the association between vitamin B12 levels and DMFT scores.

**Table 3:** Models for the association of vitamin B12 with the caries experience.

Variables	Model I^1^	Model II^2^	Model III^3^
DMFT scores	RR [95% CI]	RR [95% CI]	RR [95% CI]
Normal (>221 pmol/L)	Reference	Reference	Reference
Marginal (148–221 pmol/L)	1.07 [1.03–1.10]*	1.07 [1.03–1.11]*	1.05 [1.01–1.08]*
Deficient (<148 pmol/L)	1.10 [1.04–1.17]*	1.10 [1.03–1.16]*	1.08 [1.02–1.15]*

^1^Model I was unadjusted. ^2^Model II adjusted for age, and race/ethnicity. ^3^Model III adjusted for age, race/ethnicity, education level, and PIR. *P < 0.05.

Across all models, participants with marginal B12 levels exhibited significantly higher DMFT scores than those with normal vitamin B12 levels. In the fully adjusted model (Model III), accounting for age, race/ethnicity, education, and income, the rate ratio (RR) for marginal vitamin B12 status was 1.05 (95% CI: 1.01–1.08, *p* < 0.05). Likewise, individuals with vitamin B12 deficiency had significantly increased DMFT scores using the confounding variable model (RR: 1.08, 95% CI: 1.02–1.15, *p* < 0.05) (Table 3), suggesting an association between low vitamin B12 levels and greater overall dental disease burden.

## 4 Discussion

After adjusting for covariates, our study identified a significant relationship between lower vitamin B12 levels and higher DMFT scores. Demographic and socioeconomic factors such as sex, race/ethnicity, education, and poverty status attenuated the association of dental health outcomes and vitamin B12 levels, highlighting the multifaceted nature of dental health disparities in this population. Based on these findings, we accepted the hypothesis that lower vitamin B12 levels are associated with higher DMFT scores.

These findings largely align with previous research indicating that poor vitamin B12 status adversely affects dental health. Past studies found that vitamin B12 deficiency is associated with a greater prevalence of periodontitis and tooth loss (31). Previous studies also found that lower socioeconomic status and educational level correlate with vitamin B12 deficiency (32, 33), and our study results support the interconnectedness between low socioeconomic status, Vitamin B12 levels, and dental health. However, the present study differs from earlier research by demonstrating a direct link between vitamin B12 deficiency and DMFT scores (19, 34).

The mechanisms behind the association between vitamin B12 deficiency and increased DMFT scores may relate to vitamin B12’s role in cell regeneration, immune function, and mucosal health (35, 36), which is essential for maintaining the oral mucosa and gum tissues (37). Inadequate vitamin B12 levels can lead to impaired healing and increased susceptibility to bacterial infections which contribute to dental caries and periodontal disease (38, 39). The association between vitamin B12 deficiency and higher DMFT scores may reflect the cumulative and progressive nature of dental disease, where deficiencies in vitamin B12 contribute to damage manifested by poorer DMFT scores.

These findings illustrate the potential importance of vitamin B12 levels in dental care for older adults, particularly given this population’s higher prevalence of vitamin B12 deficiency (9, 35). Screening for vitamin B12 deficiency as part of routine dental health assessments could help identify individuals at higher risk for dental disease, enabling targeted interventions to prevent further deterioration. Potential interventions could include modifying an individual’s diet or recommending a B12 supplement (35). Another factor to consider is the disparity in dental health among socioeconomic and racial/ethnic groups (32). Addressing these disparities is important for reducing the burden of vitamin B12 deficiency and dental disease in vulnerable populations. For individuals with limited access to sufficient vitamin B12, interventions could include dietary modifications, supplementation, and increasing the consumption of vitamin B12 fortified foods (40).

### 4.1 Strengths and limitations

This study has several strengths, including its use of NHANES data, a large, nationally representative sample of older adults. Additionally, using biochemical measures of vitamin B12 levels rather than self-reported dietary intake reduces the risk of misclassification bias. The study also benefits from rigorous dental health assessments conducted by trained dental professionals using standardized NHANES protocols. However, several limitations should be acknowledged. The cross-sectional design of NHANES prevents establishing causal relationships between vitamin B12 levels and dental caries. Unaccounted variables such as diet, oral hygiene behaviors, and use of dental services might have altered the results. However, adding more covariates would increase the risk of overfitting and reduce the interpretability and stability of the model. Additionally, excluding individuals with missing data could introduce selection bias, potentially affecting the generalizability of the results. Finally, the lack of longitudinal follow-up limits the ability to assess changes in vitamin B12 levels and their impact on dental health over time.

### 4.2 Future research directions

Further investigations using longitudinal designs will be helpful to explore causality and whether changes in vitamin B12 status over time influence dental health outcomes. Interventional studies assessing the effects of vitamin B12 supplementation on dental disease progression could provide valuable insights into potential preventive strategies. While the exact pathophysiology of how B-12 affects dental health remains uncertain, its biological activity, such as the influence of vitamin B12 on immune function, salivary composition, dental enamel, oral mucosa, and oral microbiota offers mechanisms of how this nutrient affects dental health. A recent study also found an association between B-12 and periodontal disease (41) and earlier research linked low serum B12 levels with increased clinical attachment loss and subsequent tooth loss (31). Further research can also identify how chronic diseases and other micronutrient deficiencies might interact with B-12 and its impact on dental health. Finally, given the observed socioeconomic and racial disparities in vitamin B12 deficiency and dental disease burden, future work should explore targeted interventions for high-risk populations to reduce dental health inequalities in aging adults.

### 4.3 Conclusion

The study found evidence that lower vitamin B12 levels are associated with higher DMFT scores, suggesting a greater lifetime burden of dental disease among older adults with B12 deficiency or marginal levels. These findings underscore the importance of dental health when managing nutritional deficiencies in aging populations. While this study adds to the growing body of evidence on the important role of micronutrients in dental health, further research is needed to confirm this association, explore underlying mechanisms, and assess the potential benefits of vitamin B12 supplementation as a dental health preventive measure in older adults.

## Data Availability

Publicly available datasets were analyzed in this study. This data can be found at: https://wwwn.cdc.gov/nchs/nhanes/Default.aspx.
